# Long-term outcomes of ranibizumab vs. aflibercept for neovascular age-related macular degeneration and polypoidal choroidal vasculopathy

**DOI:** 10.1038/s41598-021-93899-x

**Published:** 2021-07-16

**Authors:** Ki Won Jin, Jae Hui Kim, Jun Young Park, Sang Jun Park, Kyu Hyung Park, Joo Yong Lee, Se Joon Woo

**Affiliations:** 1 Department of Ophthalmology, Seoul National University College of Medicine, Seoul National University Bundang Hospital, 173-82 Gumi-ro, Bundang-gu, Seongnam, Gyeonggi-do 13620 South Korea; 2 Department of Ophthalmology, Kim’s Eye Hospital, Seoul, South Korea; 3 Department of Ophthalmology, Asan Medical Center, University of Ulsan College of Medicine, Seoul, South Korea

**Keywords:** Medical research, Outcomes research

## Abstract

To evaluate the long-term outcomes of ranibizumab (RBZ) vs. aflibercept (AFL) in treatment-naïve eyes with typical neovascular age-related macular degeneration (nAMD) and polypoidal choroidal vasculopathy (PCV). This multicenter, retrospective, matched-cohort analysis was conducted on data  up to 4 years of follow-ups. The primary outcome was the visual acuity (VA) change from baseline. The secondary outcomes included the number of injections, proportion of eyes without a yearly injection, and the number of eyes with treatment switching. Subgroup analyses were performed for typical nAMD and PCV. Typical nAMD was defined as nAMD other than PCV. We included VA-matched 215 eyes of 209 patients (131 and 84 eyes with RBZ and AFL, respectively). The crude mean VA changes from baseline were + 6.7 vs. + 2.6, + 2.1 vs. − 0.4, − 1.3 vs. − 1.8, and − 2.2 vs. − 5.0 letters in the RBZ and AFL groups, at 1, 2, 3, and 4 years, respectively (p > 0.05). The adjusted predicted VA by linear mixed model, proportion of eyes stratified by VA, and the survival curve for significant vision loss were comparable during the 4-year follow-up (p > 0.05). The mean number of injections were similar between the RBZ and AFL groups (2.9 vs. 3.0, respectively, p = 0.692). The subgroup analysis for typical nAMD and PCV showed similar results between the groups. The visual outcomes did not differ between RBZ and AFL during 4 years with comparable numbers of injections. Our study reflects the long-term, real-world clinical practice and treatment pattern of two treatments for typical nAMD and PCV.

## Introduction

Age-related macular degeneration (AMD) is a leading cause of blindness in elderly patients in the developed world^1^. The introduction of intravitreal anti-vascular endothelial growth factor (anti-VEGF) agents lead to paradigm shifts, as they became the first-line treatment for neovascular AMD (nAMD)^2^. Current anti-VEGF agents include ranibizumab (RBZ), aflibercept (AFL), and off-label use of bevacizumab. Two phase-III pivotal trials of RBZ, MARINA and ANCHOR showed visual gains of 7.2 letters and 10.7 letters at 24 months after monthly injections, respectively^3, 4^. For AFL, the VIEW 1 and 2 pivotal trials showed non-inferiority of eight weekly injections of AFL over monthly RBZ^5, 6^. However, fixed-regimens in the real-world are associated with economic and treatment burdens for both patients and physicians. Thus, attempts have been made to reduce the number of injections and visits by reactive *pro re nata* (PRN) or proactive treat-and-extend (T&E) regimens^7–9^. To evaluate the therapeutic effect of anti-VEGF in these regimens in routine clinical practice, large-scale, long-term real-world studies are warranted. Furthermore, a head-to-head comparison of the efficacy of RBZ and AFL in the treatment of nAMD using either a clinical trial or real-world study is lacking^5, 6, 10–13^.

Polypoidal choroidal vasculopathy (PCV) is thought to be a subtype of nAMD, which is more common in younger (< 50 years) and Asian populations (25–65%)^14^. The standard treatment of PCV is anti-VEGF monotherapy or anti-VEGF combined with photodynamic therapy (PDT)^14^. Most of the current real-world studies were conducted in western countries. Thus, only a few studies examined the real-world outcomes of anti-VEGFs on the PCV subtype^15–17^. No well-designed inter-drug comparison studies for PCV have been conducted to date. Identifying the differential efficacy between the two drugs in the real-world would benefit physicians when choosing the optimal anti-VEGF agents for nAMD and PCV.

We conducted a multicenter retrospective cohort-matched study to evaluate the long-term, real-world treatment outcomes of RBZ vs. AFL in treatment-naïve eyes with typical nAMD and PCV over a 4-year period.

## Methods

### Design and settings

This study was a multicenter, retrospective, matched-cohort analysis. The data were gathered by medical chart review from real-world routine clinical databases at three different participating institutions: two tertiary referral hospitals (Seoul National University Bundang Hospital and Asan Medical Center) and one specialized eye center (Kim’s Eye Hospital). This is part of the Bundang AMD cohort study (report 4). This study adhered to the tenets of the Declaration of Helsinki and was approved by the Institutional Review Board (IRB) of the Seoul National University Bundang Hospital (IRB No. B-1910-571-102). Informed consent was waived due to the retrospective nature of the study, and the waiver was provided by the IRB of the Seoul National University Bundang Hospital.

### Patients

We enrolled treatment-naïve eyes with newly diagnosed nAMD that started treatment with either RBZ (Lucentis; Genentech, Inc., CA/Novartis, Basel, Switzerland; 0.5 mg/0.05 mL) or AFL (Eylea; Regeneron, Inc., NJ/Bayer, Leverkusen, Germany; 2 mg/0.05 mL), from March 1, 2007 to June 31, 2017. Eyes were included only when the same drug was maintained without switching for at least 1 year after the initial treatment. In South Korea, AFL became available and was funded in 2013. Therefore, only eyes that started treatment after 2013 were included for patient matching between the two treatment groups. Consequently, eyes that started treatment between January 2013 and June 2015 were included in the present study. Eyes were matched for baseline visual acuity (VA), and a matching ratio of 1:2 (RBZ to AFL) was used to maintain the maximum number of subjects as possible.

**Figure 1:**
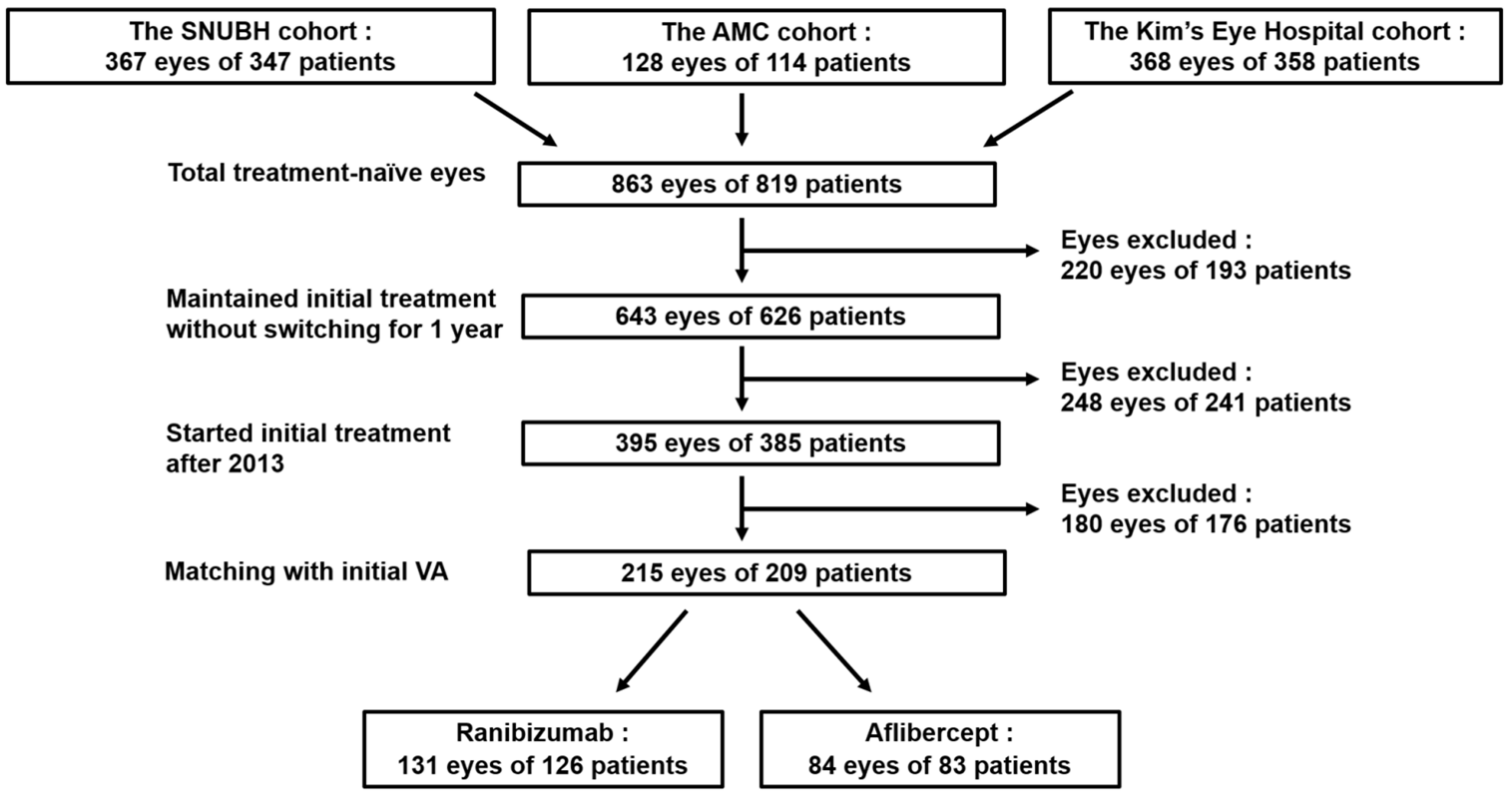
Flow charts of the enrolled population.

A total of 863 treatment-naïve eyes from 819 patients were identified. The Seoul National University Bundang Hospital, Asan Medical Center, and Kim’s Eye Hospital cohorts included 367 eyes of 347 patients, 128 eyes of 114 patients, and 368 eyes of 358 patients, respectively. From this population, 215 eyes of 209 patients (131 eyes with RBZ and 84 eyes with AFL) were finally included in the analysis after attrition by inclusion criteria and patient matching. A flow chart of the study population is presented in Fig. 1.

The treatment regimen in this study varied depending on the preference of practitioners, with either the PRN or T&E regimens adopted. The first injection date was regarded as the baseline. Labeled usage, which was reimbursed through Korean National Health Insurance, consisted of a bimonthly injection after three loading dose injections for AFL and a monthly injection after three initial injections for RBZ. For eyes with insufficient response to RBZ or AFL, the treatment could be switched by a clinician.

### Patient evaluation and grouping

In all patients, baseline ophthalmic examinations included VA measurement in decimals, dilated fundus examination, fluorescein angiography, indocyanine green angiography (ICGA), and optical coherence tomography, were performed. VA measurements with refractive error correction were conducted at every visit. The long-term longitudinal follow-up results until 4 years after the initial treatment were evaluated. The VA in decimals was converted into the Early Treatment Diabetic Retinopathy Study letter scores for arithmetic comparison. Eyes were primarily analyzed by treatment group, that is, either RBZ or AFL. For the subgroup analysis, eyes were divided into typical nAMD and PCV eyes. PCV was defined using the following diagnostic criteria: nodular hyperfluorescence of the polyps on ICGA, hypofluorescent halo around the nodules, abnormal vascular channels supplying the polyps (branching vascular networks (BVN)), and orange subretinal nodules on fundus photography corresponding to the polyps on ICGA, as diagnosed in the EVEREST study report 2^18^. nAMD other than PCV was regarded as typical nAMD, which included classic choroidal neovascularization (CNV), occult CNV, and retinal angiomatous proliferation.

### Study outcomes

The primary outcomes were the mean VA changes from baseline. Additional visual parameters, including the adjusted prediction of mean VA, the proportion of eyes stratified by VA, and the survival analysis without significant vision loss, were evaluated. VA stratification was evaluated by calculating the proportion of eyes with VA ≥ 70 letters (Snellen’s equivalent = 20/40, the threshold of driving vision in the United States) and VA ≤ 35 letters (Snellen’s equivalent = 20/200, legally blind). Significant vision loss in the survival analysis was defined as losing 10 letters from baseline at a certain point of the follow-up year. Secondary outcomes included the mean number of injections, the proportion of eyes without a yearly injection, and the number of eyes where the treatment was switched. Subgroup analyses were performed for typical nAMD and PCV. An evaluation of completion rate and a comparison between completers and non-completers were performed. Completion was defined as completing the follow-up until the end of the observation period, regardless of the yearly injection count or treatment switching.

### Statistical analysis

All analyses of the demographics and outcomes were based on the eye as the unit of analysis. A nearest-neighbor strategy-based matching of 1:2 ratio was implemented. Baseline VA was considered as the matching condition. A linear mixed-effect model was used to compensate for the loss to follow-up (LTFU)**.** The chi-square test was used to compare the categorical variables between groups. Continuous variables were compared using independent t-tests. A Kaplan–Meier survival analysis with the log-rank test was utilized to compare the cumulative probability of survival without significant vision loss over time between the groups. Patient matching and linear mixed-effect models were calculated using R software version 3.5.3 (R Project for Statistical Computing, Vienna, Austria). Analyses other than patient matching and the linear mixed-effect models were performed using SPSS software version 25.0.K (IBM Corporation, Chicago, IL, USA). A p-value of less than 0.05 was considered statistically significant.

## Results

### Study participants

The study’s baseline demographic and clinical characteristics are presented in Table 1, which were generally similar and balanced between the RBZ and AFL treatment groups. Baseline mean VA and the proportion of eyes with VA ≥ 70 letters and VA ≤ 35 letters were similar between groups. There was a significant difference in the proportion of subretinal hemorrhage (SRH). SRH was observed in 22.9% of the RBZ group and 11.9% in the AFL group (p = 0.043), and both groups showed a mean early onset of SRH after initial treatment (2.57 ± 7.04 months [RBZ] vs. 3.00 ± 9.00 months [AFL], p = 0.880). The results of the subgroup comparison for typical nAMD and PCV were well-balanced as shown in Table 1, and the proportion of eyes with hypertension was higher in the RBZ group of the typical nAMD subgroup (52.9% [RBZ] vs. 27.5% [AFL], p = 0.015).

**Table 1:** Demographic and clinical characteristics of eyes treated with ranibizumab and aflibercept.

Characteristics	Total eyes			Typical nAMD			PCV		
	Ranibizumab	Aflibercept	*p* value	Ranibizumab	Aflibercept	*p* value	Ranibizumab	Aflibercept	*p* value
Number of eyes	131 eyes of 126 patients	84 eyes of 83 patients		66 eyes of 64 patients	45 eyes of 44 patients		65 eyes of 62 patients	39 eyes of 39 patients	
Bilaterality, n (%)	5 (3.8%)	1 (1.2%)	0.617*	2 (3.0%)	1 (2.2%)	0.797*	3 (4.6%)	0 (0.0%)	0.173*
Age (years), mean ± SD	69.79 ± 8.60	70.62 ± 7.87	0.479^†^	73.85 ± 6.58	72.42 ± 7.10	0.280†	65.68 ± 8.50	68.54 ± 8.29	0.096^†^
Sex, male (%)	75 (57.3%)	50 (59.5%)	0.742*	41 (62.1%)	22 (48.9%)	0.167*	50 (76.9%)	27 (69.2%)	0.386*
DM, n (%)	18/101 (17.8%)	21/75 (28.0%)	0.108*	13/51 (25.5%)	13/40 (32.5%)	0.463*	5/50 (10.0%)	8/35 (22.9%)	0.105*
HTN, n (%)	44/101 (43.6%)	25/77 (33.3%)	0.169*	27/51 (52.9%)	11/40 (27.5%)	0.015*	17/50 (34.0%)	14/35 (40.0%)	0.572*
Typical nAMD/PCV, n	66/65	45/39	0.648*						
Baseline VA (LogMAR letter), mean ± SD	52.98 ± 21.41	52.50 ± 21.44	0.872^†^	49.76 ± 19.64	52.20 ± 20.67	0.530†	56.26 ± 22.75	52.85 ± 22.57	0.459^†^
VA ≥ 70 letters, n (%)	46 (35.1%)	28 (32.1%)	0.789*	17 (25.8%)	13 (28.9%)	0.715*	29 (44.6%)	15 (38.5%)	0.539*
VA ≤ 35 letters, n (%)	40 (30.5%)	27 (32.1%)	0.804*	26 (37.9%)	16 (35.6%)	0.803*	15 (23.1%)	11 (28.2%)	0.559*
Subretinal hemorrhage, n (%)	30 (22.9%)	10 (11.9%)	0.043*	13 (19.7%)	3 (6.7%)	0.055*	17 (26.2%)	7 (17.9%)	0.336*
Onset (months), mean ± SD	2.57 ± 7.04	3.00 ± 9.00	0.880^†^	3.69 ± 7.17	13.50 ± 19.09	0.161^†^	1.70 ± 7.03	0.00 ± 0.00	0.533^†^
**Treatment modality**									
Anti-VEGF monotherapy, n (%)	128 (97.7%)	86 (100%)	0.158*	65 (98.5%)	45 (100%)	0.407*	63 (96.9%)	39 (100%)	0.269*
Combined with PDT, n (%)	3 (2.3%)	0 (0.0%)	0.158*	1 (1.5%)	0 (0.0%)	0.407*	2 (3.1%)	0 (0.0%)	0.269*
Follow-up period (years), mean ± SD	2.59 ± 1.35	2.71 ± 1.26	0.492^†^	2.45 ± 1.38	2.69 ± 1.24	0.363^†^	2.72 ± 1.31	2.74 ± 1.29	0.938^†^
Completers of 1 year, n (%)	113 (86.3%)	76 (90.5%)	0.355*	56 (84.8%)	42 (93.3%)	0.172*	57 (87.7%)	34 (87.2%)	0.939*
Completers of 2 years, n (%)	86 (60.9%)	64 (76.2%)	0.101*	40 (60.6%)	35 (77.8%)	0.058*	46 (70.8%)	29 (74.4%)	0.693*
Completers of 3 years, n (%)	66 (50.4%)	43 (51.2%)	0.908*	30 (45.5%)	22 (48.9%)	0.722*	36 (55.4%)	21 (53.8%)	0.879*
Completers of 4 years, n (%)	57 (43.5%)	37 (44.0%)	0.938*	27 (40.9%)	19 (42.2%)	0.890*	30 (46.2%)	18 (46.2%)	1.000*

*DM* diabetes mellitus, *HTN* hypertension, *nAMD* neovascular age-related macular degeneration, *PCV* polypoidal choroidal vasculopathy, *VA* visual acuity, *LogMAR* Log minimum angle of resolution, *SRH* subretinal hemorrhage, *Anti-VEGF* anti-vascular endothelial growth factor, *PDT* photodynamic therapy.

*Pearson chi-square test.

^†^Independent t-test.

### Visual outcomes

**Figure 2:**
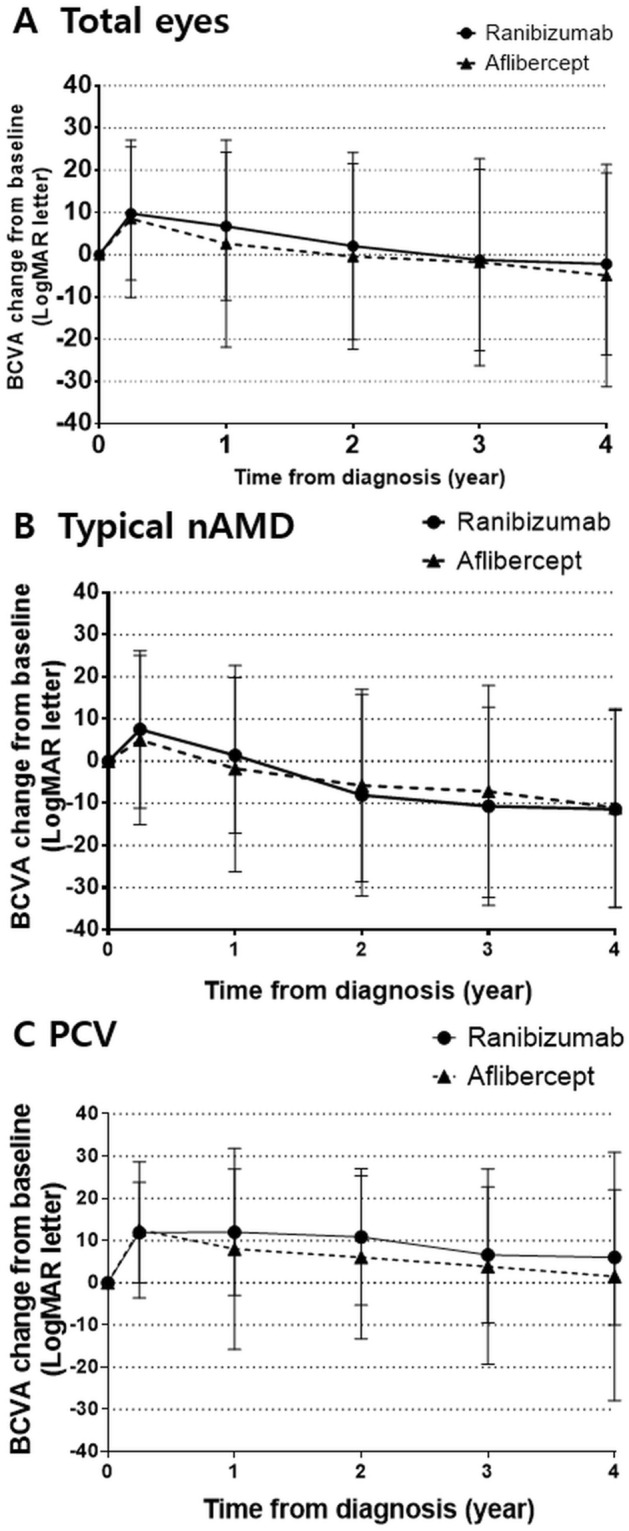
Mean change in visual acuity from baseline at each time point during the 4-year follow-up period. (**A**) Total eyes, (**B**) typical neovascular age-related macular degeneration subgroup, and (**C**) polypoidal choroidal vasculopathy subgroup.

**Figure 3:**
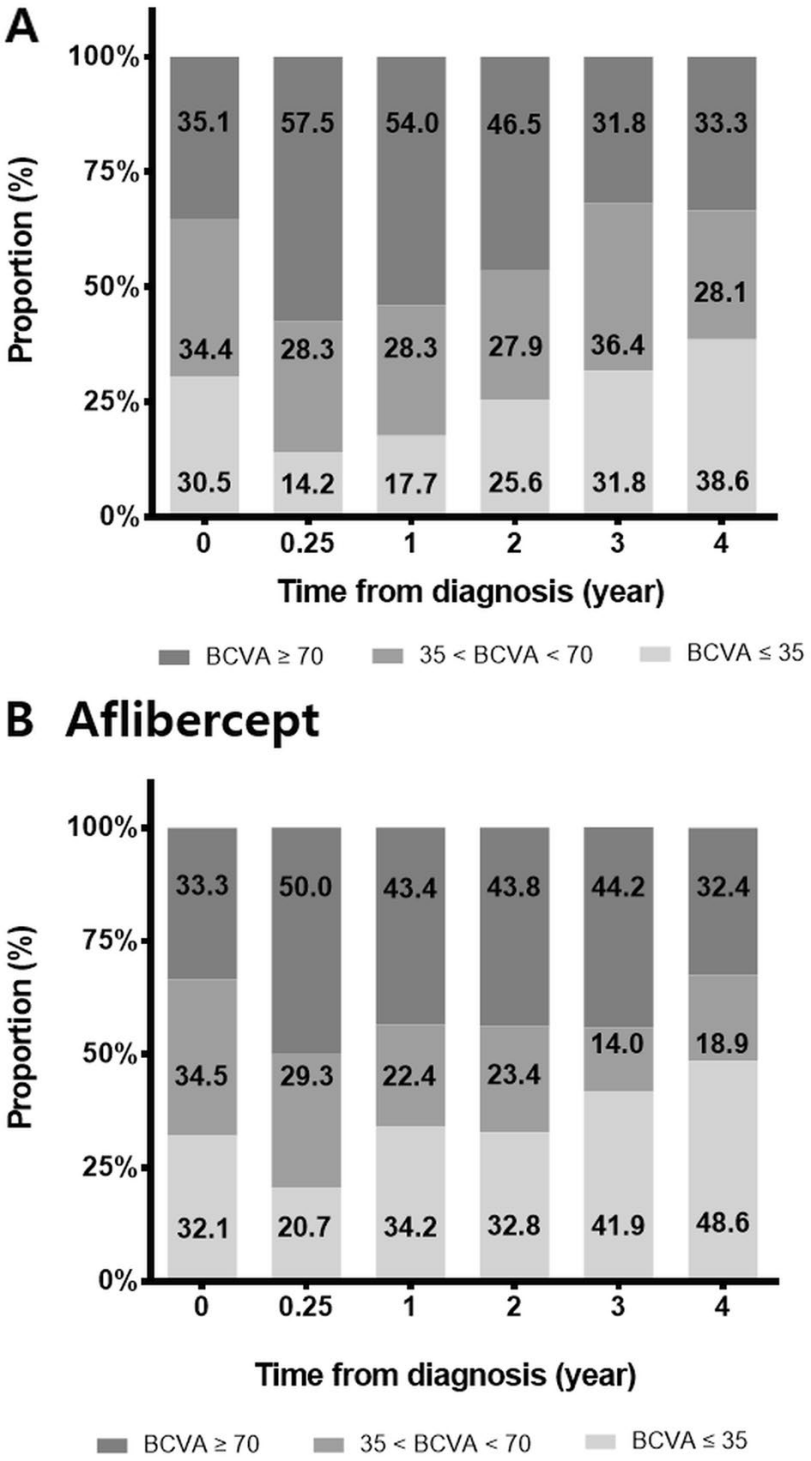
Proportion of eyes stratified by visual acuity. (**A**) ranibizumab treatment group, and (**B**) aflibercept treatment group.

The unadjusted crude mean VA changes from baseline were calculated and are shown in Fig. 2 and Table 2. The mean VA changes from baseline were + 6.7 [RBZ] vs. + 2.6 [AFL] letters at 1 year, + 2.1 [RBZ] vs. − 0.4 [AFL] letters at 2 years, − 1.3 [RBZ] vs. − 1.8 [AFL] letters at 3 years, and − 2.2 [RBZ] vs. − 5.0 [AFL] letters at 4 years (p > 0.05). The subgroup analysis for typical nAMD and PCV also showed similar comparable outcomes during the 4-year follow-up period. Eyes with typical nAMD lost − 11.4 [RBZ] vs. − 11.1 [AFL] letters at 4 years (p = 0.963), whereas eyes with PCV maintained VA above baseline for 4 years (+ 6.0 [RBZ] vs. + 1.5 [AFL] at 4 years, p = 0.492). The adjusted predicted VA values by the linear mixed model are shown together in Table 2 and were not significantly different at any point (p > 0.05). The eyes were stratified by VA and are represented in Fig. 3. The two treatment groups showed generally comparable VA outcomes, except for the proportion of eyes with VA ≤ 35 letters at 1 year, which was significantly different between the two groups (17.7% [RBZ] vs. 34.2% [AFL], p = 0.009). However, this difference was not maintained beyond 1 year (see Supplemental Table S1). The subgroup analysis for typical nAMD and PCV showed similar results. The proportion of eyes with VA ≥ 70 letters at 1 year in the PCV group showed a significant difference (75.4% [RBZ] vs. 40% [AFL], p = 0.013), but this difference was not maintained.

**Table 2:** Visual acuity changes from baseline and adjusted predictions of visual acuity by linear-mixed model.

Characteristics	Total eyes			Typical nAMD			PCV		
	Ranibizumab	Aflibercept	*p* value	Ranibizumab	Aflibercept	*p* value	Ranibizumab	Aflibercept	*p* value
**VA change from baseline (LogMAR letter), mean ± SD**									
3 months	9.71 ± 15.79	8.50 ± 18.63	0.615*	7.56 ± 18.69	5.02 ± 20.08	0.502*	11.89 ± 11.93	12.53 ± 16.13	0.821*
1 year	6.72 ± 17.52	2.62 ± 24.51	0.182*	1.39 ± 18.45	− 1.74 ± 24.46	0.472*	11.98 ± 14.95	8.00 ± 23.83	0.334*
2 years	2.05 ± 22.18	− 0.44 ± 21.95	0.497*	− 8.07 ± 23.95	− 5.77 ± 22.82	0.672*	10.85 ± 16.19	6.00 ± 19.30	0.245*
3 years	− 1.26 ± 21.48	− 1.79 ± 24.56	0.905*	− 10.67 ± 23.54	− 7.18 ± 25.14	0.611*	6.58 ± 16.09	3.86 ± 23.18	0.603*
4 years	− 2.21 ± 21.55	− 4.95 ± 26.30	0.583*	− 11.37 ± 23.42	− 11.05 ± 23.54	0.963*	6.03 ± 16.01	1.50 ± 29.42	0.492*
**Adjusted VA (LogMAR letter), mean ± SD**									
3 months	62.70 ± 2.09	61.00 ± 2.61	0.610^†^	57.47 ± 3.05	57.17 ± 3.68	0.951^†^	67.99 ± 2.62	65.48 ± 3.38	0.557^†^
1 year	59.60 ± 2.14	55.11 ± 2.65	0.187^†^	51.39 ± 3.14	50.62 ± 3.72	0.873^†^	67.87 ± 2.68	60.57 ± 3.46	0.095^†^
2 years	54.82 ± 2.28	52.36 ± 2.75	0.490^†^	42.60 ± 3.41	45.80 ± 3.87	0.535^†^	66.20 ± 2.79	60.22 ± 3.57	0.187^†^
3 years	49.99 ± 2.43	49.95 ± 3.01	0.992^†^	37.99 ± 3.68	43.49 ± 4.34	0.334^†^	61.01 ± 2.94	57.18 ± 3.82	0.427^†^
4 years	48.64 ± 2.52	46.80 ± 3.13	0.649^†^	36.81 ± 3.79	40.20 ± 4.52	0.566^†^	59.81 ± 3.07	54.21 ± 3.96	0.265^†^

*nAMD* neovascular age-related macular degeneration, *PCV* polypoidal choroidal vasculopathy, *VA* visual acuity, *LogMAR* Log minimum angle of resolution.

*Independent t-test.

^†^Linear-mixed model.

**Figure 4:**
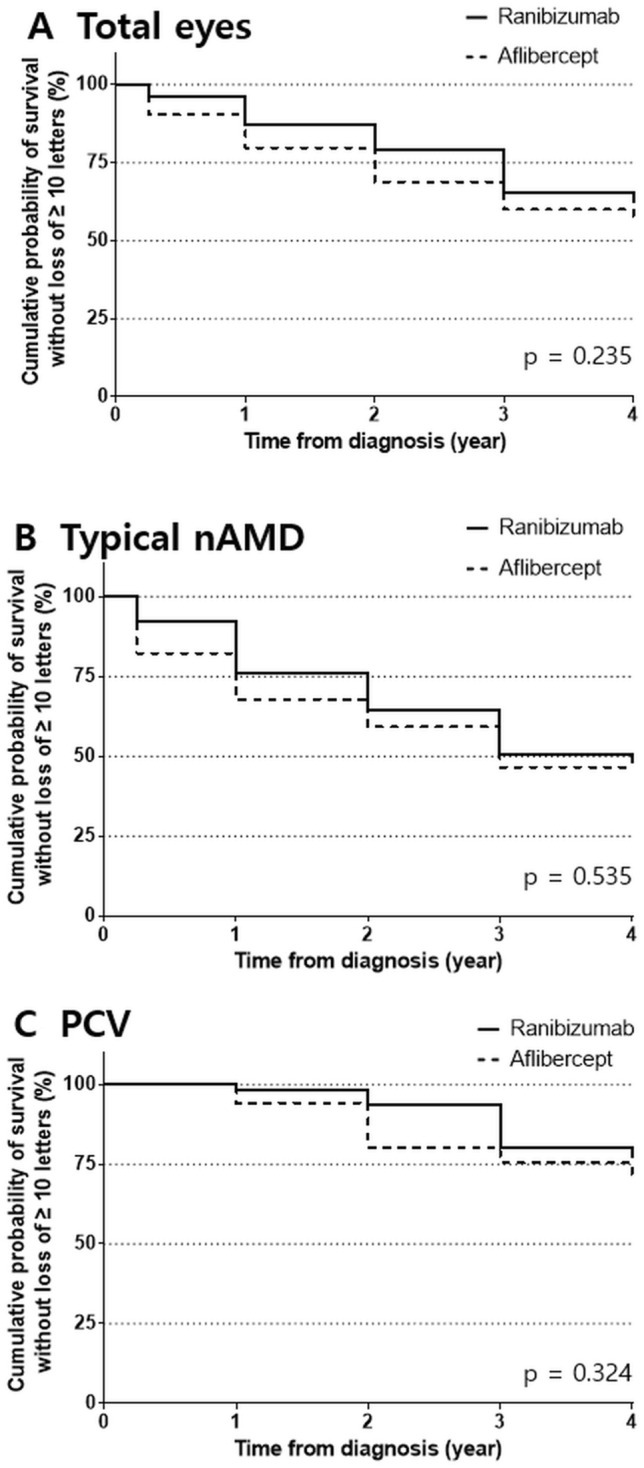
Kaplan–Meier survival curve of the cumulative probability of survival without significant vision loss. (**A**) Total eyes, (**B**) typical neovascular age-related macular degeneration subgroup, and (**C**) polypoidal choroidal vasculopathy subgroup.

The Kaplan–Meier survival curves for the cumulative probability of survival without significant vision loss (losing 10 letters) are presented in Fig. 4. The log-rank test for total eyes and the typical nAMD and PCV subgroups showed no difference between the two treatment groups (p > 0.05). The survival analysis showed that more than half of the typical nAMD eyes lost 10 letters during the 4-year follow-up period (52.42% [RBZ] vs. 53.45% [AFL]). On the other hand, only one-fourth of the PCV subgroup experienced significant vision loss (23.30% [RBZ] vs. 30.35% [AFL]).

### Number of injections

The mean number of injections was similar in both treatment groups during the follow-up period (Table 3). During the 4 years, the mean yearly injections were 2.9 ± 1.7 for the RBZ group and 3.0 ± 1.5 for the AFL group, which did not differ between the treatment groups (p = 0.692). The proportion of eyes without a yearly injection was similar between the groups during the entire study period (p > 0.05).

**Table 3:** Mean number of injections and proportion of eyes without a yearly injection.

Characteristics	Total eyes			Typical nAMD			PCV		
	Ranibizumab	Aflibercept	*p* value	Ranibizumab	Aflibercept	*p* value	Ranibizumab	Aflibercept	*p* value
**Number of Injections, mean ± SD**									
1 year	4.12 ± 1.62	4.51 ± 1.53	0.080*	4.20 ± 1.81	4.51 ± 1.53	0.342*	4.05 ± 1.41	4.51 ± 1.55	0.119*
2 years	2.00 ± 1.87	1.84 ± 1.51	0.585*	2.00 ± 2.08	1.91 ± 1.60	0.843*	2.00 ± 1.69	1.76 ± 1.43	0.527*
3 years	1.64 ± 1.90	1.51 ± 1.65	0.726*	1.73 ± 2.12	1.32 ± 1.70	0.452*	1.56 ± 1.73	1.71 ± 1.62	0.734*
4 years	1.35 ± 1.64	1.64 ± 1.92	0.716*	1.56 ± 1.78	1.21 ± 2.02	0.544*	1.17 ± 1.51	1.78 ± 1.83	0.217*
Mean yearly injections, mean ± SD	2.92 ± 1.66	3.01 ± 1.46	0.692*	3.14 ± 1.98	3.01 ± 1.59	0.701*	2.70 ± 1.24	3.01 ± 1.33	0.222*
**Proportion of eyes without a yearly injection, n (%)**									
2 years	28/85 (32.9%)	17/64 (26.6%)	0.401^†^	15/40 (37.5%)	9/35 (25.7%)	0.275^†^	13/45 (28.9%)	8/29 (27.6%)	0.903^†^
3 years	32/66 (48.5%)	19/43 (44.2%)	0.660^†^	15/30 (50.0%)	11/22 (50.0%)	1.000^†^	17/36 (47.2%)	8/21 (38.1%)	0.503^†^
4 years	29/57 (50.9%)	19/37 (51.4%)	0.964^†^	13/27 (48.1%)	12/19 (63.2%)	0.314^†^	16/30 (53.3%)	7/18 (38.9%)	0.332^†^

*nAMD* neovascular age-related macular degeneration, *PCV* polypoidal choroidal vasculopathy.

*Independent t-test.

^†^Pearson chi-square test.

### Treatment switching

Treatment switches were only reported in the RBZ treatment group. Thus, switching from RBZ to AFL was significantly more frequent (13.7% [RBZ] vs. 0% [AFL], p = 0.000). The mean follow-up period prior to switching was 2.3 ± 0.6 years. The mean VA when the treatment was switched and mean VA at 1 year after switching were 69.3 ± 9.6 and 66.9 ± 9.2 letters, respectively, and did not differ significantly (p = 0.425, paired t-test). The subgroup analysis for typical nAMD and PCV showed similar results to the total eyes (see Supplemental Table S2).

### Completion rate and comparison between completers and non-completers

The mean follow-up period was 2.6 ± 1.4 years in the RBZ group and 2.7 ± 1.3 years in the AFL group (p = 0.492). Although the completion rate of 1 year was as high as 86.3% [RBZ] vs. 90.5% [AFL], the final 4-year follow-up rates were 43.5% (RBZ) vs. 44.0% (AFL) (p > 0.05; Table 1). We compared the variables between the completers and non-completers at each time point and found that baseline VA, VA at the last follow-up, and whether they were diagnosed with typical nAMD or PCV did not differ, but non-completers after 1 year of follow-up were significantly older (p < 0.05; see Supplemental Table S3).

## Discussion

In this 4-year long-term multicenter retrospective study, the visual outcomes in the form of adjusted predictions, mean VA change from baseline, proportion of eyes stratified by VA, and survival analysis without significant vision loss were not different between the two treatment groups. The number of injections and the proportion of eyes without yearly injection also did not differ between the groups. The subgroup analysis for typical nAMD and PCV showed comparable results between the treatment groups.

The majority of current real-world studies have reported short-term 1- or 2-year outcomes^19–21^, and results of a single anti-VEGF agent^22–27^ or a mixture of anti-VEGFs without classification^28–34^. Few studies have compared two anti-VEGF drugs^10–13^, and long-term head-to-head comparative outcomes to date were limited to 3 years or shorter (Table 4)^12^. We now report the long-term, 4-year outcomes between RBZ and AFL. Furthermore, to the best of our knowledge, we report the first comparative study between treatments for PCV in an Asian population.

**Table 4:** Comparison of published real-world comparative studies between ranibizumab and aflibercept.

Study	Country	Data source	Treatment	Subjects	Numbers of subjects (eyes)	Regimen	Study period	Age of subjects (years)	Baseline VA	VA change from baseline	Mean number of injections	Conclusion
Gilles et al.^10^	Australia, New Zealand, Switzerland	FRB! registry	RBZ, AFL	Treatment-naïve eyes	394 (197 RBZ, 197 AFL)	Monthly, PRN, or T&E	12 months	81.1 [RBZ] vs. 79.9 [AFL]	58.6 [RBZ] vs. 58.9 [AFL] letters	+ 3.7 [RBZ] vs. + 4.26 [AFL] letters	8.1 [RBZ] vs. 8.0 [AFL]	Similar efficacy and treatment pattern
Lotery et al.^11^	US	Standardized US EMR system	RBZ, AFL	Excluded eyes received anti-VEGF within 6 months	7650 (3350 RBZ, 4300 AFL)	N/A	12 months	83.4 [RBZ] vs. 82.4 [AFL]	57.5 [RBZ] vs. 58.5 [AFL] letters	− 0.30 [RBZ] vs. − 0.19 [AFL] letters	6.7 [RBZ] vs. 7.0 [AFL]	Similar efficacy and treatment pattern
Rao et al.^13^	US	AAO IRIS registry	RBZ, AFL, BVZ	Eyes received anti-VEGF within 12 months were excluded	13,859 (2749 RBZ, 4387 AFL, 6723 BVZ)	N/A	12 months	81.4 [RBZ] vs. 80.5 [AFL] vs. 80.9 [BVZ]	0.54 [RBZ] vs. 0.53 [AFL] vs. 0.61 [BVZ]	− 0.053 [RBZ] vs. − 0.040 [AFL] vs. − 0.048 [BVZ] logMAR	6.5 [RBZ] vs. 6.2 [AFL] vs. 5.9 [BVZ]	Similar efficacy; fewer injections in BVZ than RBZ and AFL
Bhandari et al.^12^	Australia, New Zealand, Switzerland	FRB! registry	RBZ, AFL	Treatment-naïve eyes	965 (499 RBZ, 466 AFL)	N/A	3 years	82 [RBZ] vs. 79 [AFL]	59.9 [RBZ] vs. 58.2 [AFL] letters	+ 4.6 [RBZ] vs. + 4.5 [AFL] letters at 2 years; + 1.5 [RBZ] vs. + 1.6 [AFL] letters at 3 years	median 18 [RBZ] vs. median 18 [AFL] (total 3 years)	Similar efficacy and treatment pattern
Present study (Bundang AMD cohort study 4)	South Korea	EMR chart review from multicenter, matched-cohort	RBZ, AFL	Treatment-naïve eyes	215 (131 RBZ, 84 AFL)	PRN or T&E	4 years	69.8 [RBZ] vs. 70.6 [AFL]	53.0 [RBZ] vs. 52.5 [AFL] letters	+ 6.7 [RBZ] vs. + 2.6 [AFL] letters at 1 year; + 2.1 [RBZ] vs. − 0.4 [AFL] letters at 2 year, − 1.3 [RBZ] vs. − 1.8 [AFL] letters at 3 years; − 2.2 [RBZ] vs. − 5.0 [AFL] letters at 4 years	4.1 [RBZ] vs. 4.5 [AFL] for 1 year; Mean 2.9 [RBZ] vs. 3.0 [AFL] during 4 years	Similar efficacy and treatment pattern in nAMD and PCV

*VA* visual acuity, *EMR* electronic medical records, *RBZ* Ranibizumab, *AFL* Aflibercept, *BV*Z Bevacizumab, *PRN* pro re nata, *T&E* treatment and extend, *LogMAR* Log minimum angle of resolution.

The visual outcomes of the present study did not differ between the two treatment groups. The outcomes of our results (+ 6.7 [RBZ] vs. + 2.6 [AFL] letters at 1 year, + 2.1 [RBZ] vs. − 0.4 [AFL] letters at 2 years, respectively, p > 0.05) were comparable to the AURA study with RBZ (+ 2.4 letters at 1 year, + 0.6 letters at 2 years)^20^, the study of UK AMD EMR Users group with RBZ (+ 2 letters at 1 year, + 1 letter at 2 years)^23^, and the comparative study of a large US data set (− 0.3 [RBZ] vs. − 0.19 [AFL] letters at 1 year)^11^. However, direct comparisons must be made cautiously due to the studies focusing on different drugs and regimens. However, the meta-analysis results of 42 studies with RBZ (+ 5.0 letters at 1 year, + 3.4 letters at 2 years) and the Fight Retinal Blindness study! (FRB!) registry with mixed treatment and the T&E regimen (+ 5.3 letters at 2 years) showed better results with higher mean yearly injections^19, 35^. The 3- and 4-year outcomes of our study (− 1.3 [RBZ] vs. − 1.8 [AFL] letters at 3 years, − 2.2 [RBZ] vs. − 5.0 [AFL] letters at 4 years, p > 0.05) showed comparable results with the study using anonymized US EMR data with mixed treatment (− 3.1 letters at 3 years and − 5.2 letters at 4 years)^33^. However, a recent study with FRB! registry reported better outcomes at 3 years (+ 1.5 [RBZ] vs. + 1.6 [AFL] letters at 3 years).

The number of injections at 1 year (4.1 [RBZ] vs. 4.5 [AFL], p > 0.05) and the mean yearly injections (2.9 [RBZ] vs. 3.0 [AFL], p > 0.05) showed much lower numbers than the label-recommended dosing, but the number of injections was not different between groups. The numbers of injections were lower than previous comparative studies on nAMD with a mean number of 6.4–8.1 [RBZ] vs. 6.2–8.0 [AFL] at 1 year^10, 11, 13^, and a median number of 5 [RBZ] vs. 4 [AFL] at 2 years and 5 [RBZ] vs. 5 [AFL] at 3 years^12^. This Under-treatment may be due to the patients having worse baseline macular condition with worse baseline VA (53.0 [RBZ] vs. 52.5 [AFL] letters, p > 0.05) in the current study compared to that in other comparative studies (57.5–58.6 [RBZ] vs. 58.2–59.9 [AFL] letters), with the possible inclusion of patients with advanced lesions of geographic atrophy or disciform scar (Table 4)^10–13^. Furthermore, the effect of domestic insurance systems must be considered. The Korean National Health Insurance system restricted the number of injections to 14 injections per patient over their lifetime until November 2017. In December 2017, the insurance policy was revised, and there are no longer any limits on the number of injections a patient can receive. On the other hand, injections for eyes with VA lower than 20/200 become non-funded. The patients had been treated for at least 2.5 years of the entire 4 years as per the regulation. Although the exact impact on the injection counts remains unclear, this could have substantially impacted the results of the present study. The authors assert that differences in insurance systems must be considered when interpreting the results of real-world studies, as the AURA study discovered that the number of visits and injections and visual outcomes varied between countries^20^. However, in this study, comparable visual outcomes were achieved with substantially fewer injections, showing the characteristics of nAMD patients in South Korea, with substantial differences in demographics including younger age (69.8 [RBZ] vs. 70.6 [AFL]) than in other western studies (81.1–83.4 [RBZ] vs. 79–82.4 [AFL]) and worse baseline VA, as mentioned above.

We could not evaluate disease activity with the present study’s data. Thus, we calculated the proportion of eyes without a yearly injection. The proportion did not differ between the two treatment groups as well as in the subgroup analyses. However, eyes without a yearly injection may include stable and inactive conditions, poor response, or advanced lesions with geographic atrophy or disciform scar change. Further studies that evaluate lesion activity are needed to confirm the results of the present study.

It remains contentious as to whether PCV is a subtype of nAMD or a distinct disease entity^36^. Two large pivotal trials were conducted for the treatment of PCV. The EVEREST II study evaluated RBZ monotherapy vs. RBZ combined with PDT and found higher visual gains in the combination group at 12 months (5.1 vs. 8.3 letters)^37^. However, the results of the PLANET study reported that AFL monotherapy was non-inferior to AFL with rescue PDT up to 96 weeks (10.7 vs. 9.1 letters), and the proportion of patients requiring rescue PDT was small (17%)^38^. The real-world outcomes of the Asian population, with a higher rate of PCV occurrence, have been underrepresented^14^. Matsumiya et al. reported 2-year visual gains of + 5.7 letters with RBZ in the PCV group^15^, and Nishikawa et al. showed that long-term, 4-year results with aflibercept and vision were retained above baseline after the 4-year treatment^17^. In the present study, 48.4% of the total eyes (104 of 215 eyes) had PCV, and the mean VA change from baseline showed that VA was maintained for the entire 4 years in the PCV subgroup, on the contrary, it was below the initial values after 1 year in the typical nAMD subgroup. The survival analysis for significant vision loss also showed that half of the typical nAMD eyes lost 10 letters during the 4-year follow-up period. In contrast, only one-fourth of the PCV subgroup experienced vision loss. Recent real-world outcomes with the FRB! registry compared anti-VEGF monotherapy with a combination of anti-VEGF and PDT and found that the combination group showed larger vision gains with fewer injections^16^. Only two patients in the PCV subgroup received PDT in this study, and we were, therefore, unable to compare the treatment modalities. Our data shows that anti-VEGF monotherapy is the mainstream treatment for PCV in South Korea. Further studies should be conducted to find the best treatment option for Asian people with PCV.

In this study, treatment switches were only reported in the RBZ treatment group, from RBZ to AFL (18 eyes, 13.7%). The rate of switching treatment is comparable to the results of previous studies (12.5, 15%)^10–12^, and eyes that switched treatment did not show a VA difference after 12 months, as previously reported by Barthelmes et al. and Chakravarthy et al.^39, 40^. We contemplate that this one-way result was due to the effect of newly introduced drugs and RBZ-refractory cases. However, the possible effect of practitioners preferring AFL for poor response eyes could not be ruled out. The results of the report that AFL further inhibits VEGF B and placental growth factor, as well as VEGF A, might have affected the drug choice^41^.

The LTFU rate in our study was comparable to the results of observational reports^42^. The LTFU results were similar between the two treatments and in the typical nAMD and PCV subgroups. Non-completers after 1 year were significantly older than completers (p < 0.05), and we believe that the inability to visit clinics and high mortality and comorbidity rates in older patients may contribute to LTFU. A previous study by Lotery et al. also reported similar results that discontinuing eyes were older, although they also found low baseline VA in non-completers^11^. Bhandari et al. reported that reasons for discontinuation were not due to poor outcomes in most cases^12^. Many previous studies used the last observation carried forward (LOCF) method, which carries the latest observed value of non-completers to the end, to deal with LTFU^10, 12, 43, 44^. We concluded that the LOCF method is not applicable in the current study and could over- or under-estimate the outcomes because of the high proportion of LTFU after the completion of 1 year. Instead, we adopted a mixed-effects regression model to make full use of the data of non-completers.

This study has several limitations. The present study was retrospective and non-randomized in design, which can lead to selection bias. This study has lower internal validity than randomized controlled trials by nature, and practitioners’ personal preferences could affect the initial drug choice and treatment regimen. Additionally, the results of the present study could be affected by the domestic insurance policy. However, our study appears to reflect the long-term, real-world clinical practice in South Korea and may be used as a clinical management resource. Further limitations include a high proportion of LTFU after 1 year; however, this is inevitable in real-world studies. In addition, the number of visits, lesion size and activity, reasons for SRH occurrence, initial drug choice, treatment discontinuation, and switching treatment could not be evaluated using the data collected as part of this study. Future well-designed studies with larger cohorts are warranted to validate the results of the present study.

In conclusion, the visual outcomes did not differ between RBZ and AFL in the treatment of treatment-naïve eyes with nAMD and PCV over a 4-year period. The number of injections and the proportion of eyes without a yearly injection were also not different between the groups. The subgroup analysis for typical nAMD and PCV showed comparable results between the treatment groups. Our study likely reflects the long-term, real-world clinical practice and treatment patterns in South Korea and compares the outcomes of two treatments for typical nAMD and PCV.

## Supplementary Information

Supplementary Table S1.

Supplementary Table S2.

Supplementary Table S3.

## Data Availability

The datasets generated during and/or analysed during the current study are available from the corresponding author on reasonable request.
