# Supplementary material for: Long-term outcomes of ranibizumab vs. aflibercept for neovascular age-related macular degeneration and polypoidal choroidal vasculopathy
Source: Sci Rep. 2021 Jul 16;11:14623. doi: 10.1038/s41598-021-93899-x (PMC8285484; doi:10.1038/s41598-021-93899-x)
Supplement: Supplementary file 1 — Supplementary Table S1. [file 41598_2021_93899_MOESM1_ESM.docx]

**Supplemental Table 1. The Proportion of Eyes Stratified by Visual Acuity**

| **Characteristics** | **Total Eyes** | | | **Typical nAMD** | | | **PCV** | | |
| --- | --- | --- | --- | --- | --- | --- | --- | --- | --- |
|  | **Ranibizumab** | **Aflibercept** | ***p* value** | **Ranibizumab** | **Aflibercept** | ***p* value** | **Ranibizumab** | **Aflibercept** | ***p* value** |
| **VA** **≥ 70 letters, n (%)** |  |  |  |  |  |  |  |  |  |
| **Baseline** | 46 / 131 (35.1%) | 28 / 84 (33.3%) | 0.789* | 17 / 66 (25.8%) | 13 / 45 (28.9%) | 0.715* | 29 / 65 (44.6%) | 15 / 39 (38.5%) | 0.539* |
| **3 months** | 73 / 127 (57.5%) | 41 / 82 (50.0%) | 0.289* | 30 / 64 (46.9%) | 19 / 44 (43.2%) | 0.705* | 43 / 63 (68.3%) | 22 / 38 (57.9%) | 0.292* |
| **1 year** | 61 / 113 (54.0%) | 33 / 76 (43.4%) | 0.154* | 18 / 56 (32.1%) | 16 / 42 (38.1%) | 0.540* | 43 / 57 (75.4%) | 17 / 34 (50.0%) | 0.013* |
| **2 years** | 40 / 86 (46.5%) | 28 / 64 (43.8%) | 0.737* | 10 / 40 (25.0%) | 13 / 35 (37.1%) | 0.255* | 30 / 46 (65.2%) | 15 / 29 (51.7%) | 0.245* |
| **3 years** | 21 / 66 (31.8%) | 19 / 43 (44.2%) | 0.190* | 6 / 30 (20.0%) | 8 / 22 (36.4%) | 0.189* | 15 / 36 (41.7%) | 11 / 21 (52.4%) | 0.433* |
| **4 years** | 19 / 56 (33.9%) | 12 / 33 (32.4%) | 0.928* | 6 / 27 (22.2%) | 4 / 19 (21.1%) | 0.925* | 13 / 30 (43.3%) | 8 / 18 (44.4%) | 0.940* |
| **VA** **≤ 35 letters, n (%)** |  |  |  |  |  |  |  |  |  |
| **Baseline** | 40 / 131 (30.5%) | 27 / 84 (32.1%) | 0.804* | 25 / 66 (37.9%) | 16 / 45 (35.6%) | 0.803* | 15 / 65 (23.1%) | 11 / 39 (28.2%) | 0.559* |
| **3 months** | 18 / 127 (14.2%) | 17 / 82 (20.7%) | 0.215* | 11 / 64 (17.2%) | 11 / 44 (25.0%) | 0.322* | 7 / 63 (11.1%) | 6 / 38 (15.8%) | 0.496* |
| **1 year** | 20 / 113 (17.7%) | 26 / 76 (34.2%) | 0.009* | 14 / 56 (25.0%) | 18 / 42 (42.9%) | 0.062* | 6 / 57 (10.5%) | 8 / 34 (23.5%) | 0.096* |
| **2 years** | 22 / 86 (25.6%) | 21 / 64 (32.8%) | 0.333* | 16 / 40 (40.0%) | 16 / 35 (45.7%) | 0.618* | 6 / 46 (13.0%) | 5 / 29 (17.2%) | 0.617* |
| **3 years** | 21 / 66 (31.8%) | 18 / 43 (41.9%) | 0.285* | 15 / 30 (50.0%) | 12 / 22 (54.5%) | 0.746* | 6 / 36 (16.7%) | 6 / 21 (28.6%) | 0.288* |
| **4 years** | 22 / 57 (38.6%) | 18 / 37 (48.6%) | 0.256* | 14 / 27 (51.9%) | 11 / 19 (57.9%) | 0.685* | 8 / 30 (26.7%) | 7 / 18 (38.9%) | 0.251* |

nAMD = Neovascular age-related macular degeneration, PCV = Polypoidal choroidal vasculopathy, VA = Visual acuity

* : Pearson chi-square test,
