# Supplementary material for: Long-term outcomes of ranibizumab vs. aflibercept for neovascular age-related macular degeneration and polypoidal choroidal vasculopathy
Source: Sci Rep. 2021 Jul 16;11:14623. doi: 10.1038/s41598-021-93899-x (PMC8285484; doi:10.1038/s41598-021-93899-x)
Supplement: Supplementary file 2 — Supplementary Table S2. [file 41598_2021_93899_MOESM2_ESM.docx]

**Supplemental Table 2. The Eyes that Switched Treatment**

| **Characteristics** | **Total Eyes** | | | **nAMD** | | | **PCV** | | |
| --- | --- | --- | --- | --- | --- | --- | --- | --- | --- |
|  | **Ranibizumab** | **Aflibercept** | ***p* value** | **Ranibizumab** | **Aflibercept** | ***p* value** | **Ranibizumab** | **Aflibercept** | ***p* value** |
| **Switching, n (%)** | 18 / 131 (13.7%) | 0 / 86 (0%) | 0.000* | 5 / 66 (7.6%) | 0 / 45 (0%) | 0.059* | 13 / 65 (20.0%) | 0 / 39 (0%) | 0.003* |
| **Mean follow-up periods prior to switching (years), mean ± SD** | 2.33 ± 0.59 |  |  | 2.40 ± 0.55 |  |  | 2.31 ± 0.63 |  |  |
| **Mean VA at switching (LogMAR letter), mean ± SD** | 69.28 ± 9.58 |  |  | 69.60 ± 7.80 |  |  | 69.15 ± 10.48 |  |  |
| **Mean VA at 1 year after switching (LogMAR letter), mean ± SD** | 66.93 ± 9.16 |  | 0.425† | 65.40 ± 9.45 |  | 0.325† | 67.70 ± 9.43 |  | 0.570† |

nAMD = Neovascular age-related macular degeneration, PCV = Polypoidal choroidal vasculopathy, VA = Visual acuity

* : Pearson chi-square test, † : Paired t-test
