# Supplementary material for: Long-term outcomes of ranibizumab vs. aflibercept for neovascular age-related macular degeneration and polypoidal choroidal vasculopathy
Source: Sci Rep. 2021 Jul 16;11:14623. doi: 10.1038/s41598-021-93899-x (PMC8285484; doi:10.1038/s41598-021-93899-x)
Supplement: Supplementary file 3 — Supplementary Table S3. [file 41598_2021_93899_MOESM3_ESM.docx]

**Supplemental Table 3. Comparison between Completers and Non-completers**

| **Characteristics** | **Completers** | **Non-completers** | **p value** | |
| --- | --- | --- | --- | --- |
| **Number of eyes, n (%) (Total 214 eyes)** |  |  |  |  |
| **Completion of 1 year** | 188 (87.9%) | 26 (12.1%) |  |  |
| **Completion of 2 years** | 149 (60.6%) | 65 (30.4%) |  |  |
| **Completion of 3 years** | 109 (50.9%) | 105 (49.1%) |  |  |
| **Completion of 4 years** | 94 (43.9%) | 120 (56.1%) |  |  |
| **Age (years), mean ± SD** |  |  |  |  |
| **Completion of 1 year** | 70.09 ± 7.98 | 70.35 ± 10.73 | 0.884* |  |
| **Completion of 2 years** | 69.26 ± 8.15 | 72.09 ± 8.49 | 0.022* |  |
| **Completion of 3 years** | 68.56 ± 8.02 | 71.74 ± 8.39 | 0.005* |  |
| **Completion of 4 years** | 68.76 ± 8.16 | 71.19 ± 8.35 | 0.034* |  |
| **Baseline VA (LogMAR letter), mean ± SD** |  |  |  |  |
| **Completion of 1 year** | 53.00 ± 21.45 | 50.38 ± 20.95 | 0.560* |  |
| **Completion of 2 years** | 53.47 ± 20.59 | 50.88 ± 23.09 | 0.415* |  |
| **Completion of 3 years** | 51.62 ± 20.24 | 53.78 ± 22.50 | 0.461* |  |
| **Completion of 4 years** | 50.99 ± 20.73 | 54.01 ± 21.83 | 0.306* |  |
| **VA at prior follow-up periods** |  |  |  |  |
| **Completion of 1 year** | 53.00 ± 21.45 | 50.38 ± 20.95 | 0.560* |  |
| **Completion of 2 years** | 59.16 ± 22.95 | 56.13 ± 25.97 | 0.472* |  |
| **Completion of 3 years** | 54.52 ± 25.01 | 54.63 ± 29.56 | 0.983* |  |
| **Completion of 4 years** | 49.78 ± 27.49 | 52.53 ± 26.44 | 0.718* |  |
| **Typical nAMD / PCV** |  |  |  |  |
| **Completion of 1 year** | 98 / 90 | 13 / 13 | 0.839† |  |
| **Completion of 2 years** | 75 / 74 | 36 / 29 | 0.497† |  |
| **Completion of 3 years** | 52 / 57 | 59 / 46 | 0.214† |  |
| **Completion of 4 years** | 46 / 48 | 65 / 55 | 0.447† |  |

VA = Visual acuity, LogMAR = Log Minimum angle of resolution, nAMD = Neovascular age-related macular degeneration, PCV = Polypoidal choroidal vasculopathy

* : Independent t-test, † : Pearson chi-square test
